# Physiological Differences in Sebum Composition in Regularly Menstruating Healthy Women

**DOI:** 10.1111/1346-8138.17908

**Published:** 2025-08-28

**Authors:** Hiu Fung Lau, Vivian Poon, Alessia Cavallo, Grazia Bottillo, Miriam Maiellaro, Ying Xu, Helen Zhao, Enrica Flori, Christos C. Zouboulis, Mauro Truglio, Federico Marini, Emanuela Camera

**Affiliations:** ^1^ Procter and Gamble International Operations SA SG Branch Singapore Singapore; ^2^ Laboratory of Cutaneous Physiopathology and Integrated Center of Metabolomics Research San Gallicano Dermatological Institute – IRCCS Rome Italy; ^3^ Procter and Gamble Company, Mason Business and Innovation Center Mason Ohio USA; ^4^ Department of Dermatology, Venereology, Allergology and Immunology, Staedtisches Klinikum Dessau Brandenburg Medical School Theodor Fontane and Faculty of Health Sciences Brandenburg Dessau Germany; ^5^ Department of Chemistry University of Rome “La Sapienza” Rome Italy

**Keywords:** free fatty acids, lipidomics, menstrual cycle, squalene, triglycerides, wax esters

## Abstract

Sex hormones regulating the menstrual cycle influence sebaceous gland cell lipogenesis and the feeling of skin oiliness or dryness on the face. The aim of this study was to elucidate sebaceous lipogenesis in females during the menstrual cycle and define their facial sebum composition. Sebum was sampled from cheeks and foreheads in 38 Chinese women, 19 with sebometry ≤ 70 μg/cm^2^ (low sebometry group, LS), and 19 with sebometry ≥ 150 μg/cm^2^ (high sebometry group, HS), in the ovulation phase (OP) and in the early luteal phase (ELP). In addition, the follicular phase (FP) and the late luteal phase (LLP) were examined within the HS group. Sebum lipid classes were quantified by GCMS and LCMS. The HS skin type was characterized by presenting more sebum lipids on the cheeks and the forehead than the LS skin type, respectively. In the HS subgroup, multivariate analysis of forehead sebum data was applied to the amounts assessed at FP, OP, ELP, and LLP. Our data detected a fluctuation of facial sebogenesis during the menstrual cycle.

## 1 Introduction

Sebum takes part in the skin surface lipids (SSL) film in body areas enriched in sebaceous glands (SGs), such as the face [1, 2]. Sebum is manufactured in the SG cells, which are the active site of lipid synthesis; sebum then egresses to the skin surface following holocrine secretion [3]. The response of the SG to various hormonal stimuli is key in the regulation of sebum secretion as witnessed by the transition of the SSL occurring at puberty [4, 5]. In post‐pubertal humans, SSL contains diverse proportions of sebaceous and epidermal lipids depending on the body regions [6, 7]. The differentiation process and turnover of sebocytes in the SG are determinants of the sebum outflow [8, 9, 10, 11, 12].

Healthy people excrete sebum at a rate approximately 1–2 μg/cm^2^/min. Sebostasis or dry skin is defined for sebum secretion rate (SER) below 0.5–1 μg/cm^2^/min; in contrast, optimal seborrhea is observed for SER values between 2 and 4 μg/cm^2^/min [13].

Sebum forms approximately 90% of the SSL and is a mixture of highly hydrophobic lipids, i.e., triglycerides (TGs, 30%–50%), wax esters (WEs, 20%–30%), and squalene (12%–20%), and mildly non‐polar lipids, i.e., free fatty acids (FFAs, 10%–30%). The percentage of squalene tends to be higher in females [14]. In contrast, cholesterol and its derivatives, i.e., cholesterol esters (CEs), originate predominantly from epidermal keratinocytes [15]. Modification of sebum secretion and composition is characteristic of dermatoses affecting the pilosebaceous unit [16, 17, 18, 19].

Both defective and excessive sebum secretion contribute to sensitive skin, which is defined as a subjective cutaneous hyper‐reactivity to different stimuli [20, 21]. Sebum levels present profound inter‐individual differences depending on gender, environment, diet, and the inherent features of skin [22]. Constitutively oily and non‐oily (ranging from dry to normal) skin types present high and intermediate to low seborrhea, respectively, independently of superimposed dermatological conditions [23].

The SG bears a constellation of receptors that impact the sebaceous secretion [23]. Expression levels and functionality of such receptors may play a significant role in determining the SG's overall activity [24, 25, 26]. Especially, the effects of estrogen on the skin have been investigated in association with the postmenopausal estrogen decline and hormone replacement studies [26]. A specific fluctuation in female hormones can be important determinants of skin conditions during the menstrual cycle [5, 20, 27, 28, 29, 30]. Regularly menstruating healthy women present different metabolic and lipidomic biomarkers in plasma across menstrual cycle phases [31]. Menstrual phases may be differently associated with dry or oily skin, while exacerbation and amelioration of skin symptoms are frequently reported coincidentally with female hormone fluctuation [30].

To gain insights in the association between sebum composition and hormone fluctuation in physiological conditions of opposite skin types, with lipidomics approaches, we analyzed sebum sampled across the menstrual cycle from the forehead and the cheek areas of healthy Chinese individuals by lipidomics approaches. The primary objective of this study was to investigate the sebum lipid compositions associated with two major stages of the menstrual cycle, namely the ovulation phase (OP) and the early luteal phase (ELP), which associate with optimal perception and discomfort of facial skin, respectively [10, 26, 27, 30, 31]. This was pursued in women with low and high sebum production.

## 2 Materials and Methods

### 2.1 Study Design

The study was approved by the Institutional Review Board and performed in agreement with the Declaration of Helsinki principles. The informed consent was undersigned by all participants before participation. The study involved Chinese women 24–29 years old who lived in the urban areas in the past 10 years with self‐declared cycle duration of 26–30 days. Exclusion criteria were smoking habits, acne, scarring, visible wrinkles, poor texture, intake of oral supplements, and medications. Additional inclusion and exclusion criteria are reported in Appendix S2.

Participants in the study recorded the bleeding day in the 2 months preceding the collection of menstrual phase records and sebum samples. The ovulation test was monitored by performing the ovulation test every day starting from day 8 until positive results were observed. The test was stopped 2 days after the first negative result was recorded.

Thirty‐eight subjects were selected among the eligible participants based on the regular menstrual cycle. The Sebumeter (Courage and Khazaka, Cologne, Germany) was used to categorize the subjects as having low (LS, ≤ 70 μg/cm^2^) or high (HS, ≥ 150 μg/cm^2^) sebum secretion as a result of sebum accumulation overnight on their foreheads. The LS and HS groups each consisted of 19 subjects.

### 2.2 Chemicals and Reagents

Methanol, isopropyl alcohol, acetone, and ethanol were of LCMS grade and were purchased from Merck (Darmstadt, Germany). LCMS grade ammonium formate was purchased from Fluka (Buchs, St. Gallen, Switzerland). Authentic free fatty acids (FFAs), fatty alcohols (FAOHs), squalene, cholesterol, and vitamin E were purchased from different suppliers as reported in Appendix S1. Deuterated cholesterol‐2, 2, 3, 4, 4, 6‐d_6_ (d6Cholesterol) and glyceryl‐d_5_‐trihexadecanoate (d5TG 48:0) were purchased from C/D/N Isotopes Inc., Pointe‐Claire, QUE, Canada. (6E, 10E, 14E, 18E)‐2, 6, 10, 15, 19, 23‐Hexamethyl‐2, 6, 10, 14, 18, 22‐tetracosahexaene‐d_6_ (d6Squalene) was purchased from Toronto Research Chemicals (TRC) North York, ONT Canada. [^2^H]17‐C18:1 (d17C18:1) was purchased from Cayman Chemical Company, Ann Arbor, MI, USA.

### 2.3 Sebum Sampling

Sebum samples were collected with a procedure modified from Camera E et al. [32]. Briefly, after washing the face with standardized cleanser, isopropanol wipes were used to remove surface sebum. This was confirmed using the Sebumeter (reading under 10 μg/cm^2^). Cuderm S100 Sebutape patches (28.6 mm × 19.1 mm) were applied to the left and right sides of the central line of foreheads and to the left and right cheeks for 30 min. Cleaned forceps were used to peel off the patches, which were folded into disposable tubes and stored at −80°C freezer until analysis. Sebum was collected every 2 days. Samples corresponding to the follicular phase (FP), ovulatory phase (OP, early luteal phase ELP), and late luteal phase (LLP) were processed for sebum lipid analyses, as illustrated in the Figure S1.

### 2.4 Sample Preparation

To extract study samples and blank tapes, enough clean glass tubes were pre‐loaded with 200 μL of the internal standard (iSTD) mixture containing d6Cholesterol, d6Squalene, d17C18:1, and d5TG 48:0 and stored at −20°C until use. Sebum lipids were extracted from two tapes as previously reported by Camera et al. [32]. Briefly, extraction was performed in ethanol containing 0.025% of butylhydroxytoluene (BHT) to prevent oxidation, followed by clean‐up with ethyl acetate. The extract was dissolved in 500 μL of acetone/methanol/isopropanol (AMI mixture) 40/40/20 v/v/v. Four aliquots of 100 μL each were transferred to amber glass, dried, and stored at −80°C until use.

The quality control (QC) samples were prepared by pooling 20 μL from the left volume of each study sample. Equal volumes of 100 μL of the QC pool were dispensed in amber glass vials and evaporated to dryness. The QC aliquots were stored at −80°C until use. Blank samples were prepared upon extraction of two blank tapes with the same protocol applied to sampling tapes.

To perform the GCMS and LCMS analyses detailed below, the dry aliquots from the sebum samples, QC, and blank samples were brought to room temperature in dim light and re‐dissolved in 200 μL of AMI mixture.

### 2.5 GCMS

Gas chromatography coupled to electron ionization mass spectrometry (GCMS) in dual scan‐selected ion monitoring was employed to quantitate target compounds in the sebum. Samples were analyzed with a GC 7890A coupled to the MS 5975 VL analyzer (Agilent Technologies, Santa Clara, CA, USA). Quantitative analysis of squalene, cholesterol, saturated FAs (SFAs), monounsaturated FAs (MUFAs), and FAOHs was performed by a GCMS method as previously reported [33, 34]. Briefly, 10 μL of the dissolved sebum extract was dried under nitrogen and derivatized with 50 μL BSTFA added with 1% trimethylchlorosilane (TCMS) in pyridine. To generate the trimethylsilyl (TMS) derivative of cholesterol, FFAs, and FAOHs, the reaction was carried out at 60°C for 60 min [35]. GC separation was performed with the 30 m–0.250 (i.d.) GC DB‐5MS UI fused silica column (Agilent Technologies), chemically bonded with a 5% diphenyl 95% dimethylpolysiloxane cross‐linked stationary phase (0.25 mm film thickness). Helium was used as the carrier gas. Samples were acquired in scan mode by electron impact (EI) MS.

### 2.6 LCMS

The chromatographic apparatus consisted of a 1200 series rapid resolution HPLC (Agilent Technologies), equipped with a degasser, a binary pump, an autosampler, and a thermostated column compartment from the same manufacturer. For the rapid resolution reversed phase HPLC (RR‐RP‐HPLC) separation, two columns were connected in series. The first one was a Zorbax SB‐C8 rapid resolution cartridge 2.1 × 30 mm 3.5 μm p.s. (Agilent Technologies) and the second one was a Zorbax SB‐C8 rapid resolution HT 2.1 × 100 mm 1.8 μm p.s. with a maximal operational backpressure at 600 Bar (from the same manufacturer). Sebum samples, blanks, QCs, and authentic standards were eluted with a binary gradient of (A) 5 mM ammonium formate in water and (B) methanol/isopropanol 95/5 v/v. The mobile phases were filtered through 0.45 μm glass filters and continuously degassed under vacuum. The elution program was as follows: 0–1 min 70% B, 20 min 99% B, 20–32 min 99% B, 34 min 100% B, 34–44 min 100% B, 56 min 70% B. A post run of 4 min at 70% B was included. The flow rate was maintained at 0.25 mL/min during the entire HPLC run and post‐run time (4 min). The column was thermostated at 60°C. The injection volume was 1 μL. The injector needle was washed with the mobile phase in the wash port during the HPLC runs. The eluent outlet was connected to two different MS analyzers for the detection and characterization.

Accurate mass measurements were conducted with a G6220A series time‐of‐flight mass spectrometer (TOF‐MS, Agilent Technologies) equipped with an electrospray ionization (ESI) interface operating in the positive ion mode. Analytes eluted from the LC system were introduced into the TOF‐MS apparatus at the operating chromatographic flow rate (see chromatographic conditions). Nitrogen was used as the nebulizing and desolvation gas. The temperature and the flow of the drying gas were 350°C and 10 L/min, respectively. The capillary and the cone voltage were 4000 and 60 V, respectively. Scan mode TOF mass spectra were acquired in the positive ion mode by using the TOF at 10 000 mass resolving power for scans over the range from m/z 100 to m/z 1200. To enhance accurate mass measurement for the ion species, a reference solution of two compounds with m/z 121.050873 and 922.009798, respectively, was vaporized in continuum in the spray chamber by means of a separate nebulizer.

### 2.7 Data Analysis

In GCMS, calibration curves were built against the deuterated iSTD d6Cholesterol, d6Squalene, and d17C18:1, and results were reported as μg per tape of individual FFAs, FAOHs, squalene, and cholesterol. Vitamin E levels were expressed in ng per tape. In LCMS, calibration curves were built with authentic compounds against the iSTD d5TG 48:0. Amounts of individual TGs, DGs, and WEs were pooled to derive total TGs, total DGs, and total WEs, respectively. The raw data acquired by both GCMS and LCMS were analyzed by the quantitative software (Mass Hunter Quantitative Analysis for GCMS—B.07.00, Agilent Technologies). Data were analyzed using the statistical and data analysis solutions XLSTAT 2020.7.2 (Addinsoft, New York, NY, USA) and MatLab (version 8.6.0 release R2015b; The Mathworks, Natick, MA, USA). Continuous variables were represented as average values with standard deviation (SD) or median values with confidence intervals. Student's *t* test or ANOVA were used for comparisons between groups. Spearman's coefficient (*R*) was used to measure the correlation between two quantitative variables. Differences were considered statistically significant with *p* ≤ 0.05. To evaluate whether there could be a significant effect of the controlled factors in the experimental design on the multivariate chromatographic profiles, due to the impossibility of using classical multivariate analysis of variances with highly correlated descriptors, data were processed using ANOVA‐simultaneous component analysis (ASCA) [36], as described in the Appendix S3.

## 3 Results

### 3.1 Profiles of Sebum Lipids Distribution in High and Low Sebometry Groups

The distribution of FFAs, FAOHs, cholesterol, squalene, DGs, TGs, WEs, and vitamin E levels in sebum were compared between the HS and LS groups by pooling the quantitative data, expressed as μg/tape for all lipids, except vitamin E that was expressed as ng/tape, from both cheek and forehead areas and both the phases, OP and ELP (Table S1A). FFAs were grouped according to their subclass as indicated in Appendix S1, and indexes of monounsaturation were given by the ratios C16:1/C16:0, C17:1/C17:0, and C18:1/C18:0. Most of the sebum lipids and the MUFA to SFA ratios presented significantly higher levels in HS, consistent with the inherently greater SG activity as well as higher SG density of this skin type compared to the LS skin [37, 38, 39].

**FIGURE 1:**
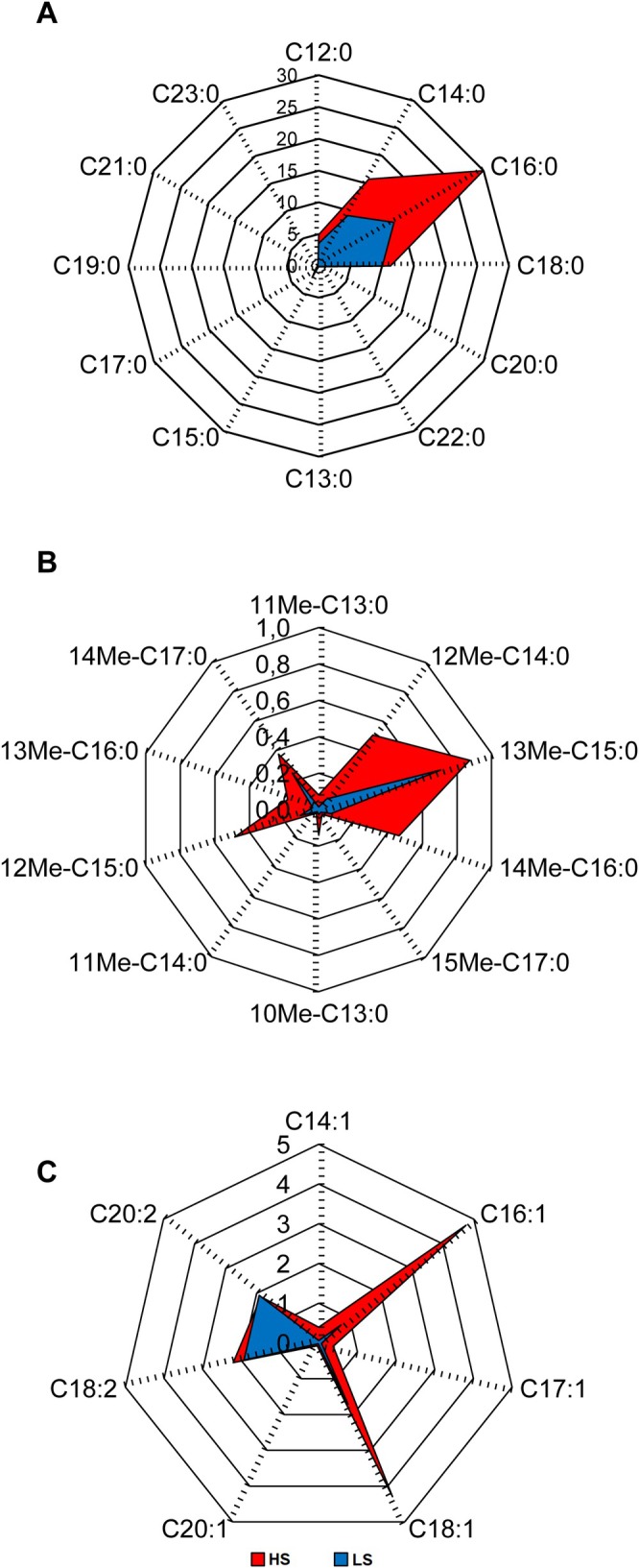
Radar diagrams depicting the distribution of straight (A), branched (B), and unsaturated (C) FFAs amounts (μg/tape) in sebum from high sebometry (HS) and low sebometry (LS) groups. Each individual axis has been arranged radially around a point. The value of each FFAs analyzed is represented by a specific node. Data values scale is reported in the principal axes: The ‘zero’ corresponds to the center of the graphic; the greater the distance from the center, the greater the quantity. The profiles of abundance of FFAs are depicted in red and blue for the HS and LS skin groups, respectively. The amounts of individual lipids and the *p*‐values of differences are reported in Table S1A.

To visualize FFAs distribution trend in HS and LS skin, profiles of FFAs absolute abundance (μg/tape) were depicted in Figure 1. As expected, straight chain FFAs with even C‐number, i.e., C12:0, C14:0, C16:0, and C18:0, were the prominent FFAs in sebum in both HS and LS groups, with the abundance order C16:0 > C14:0 > C18:0 > C12:0 (Figure 1A). Among the terminally branched FFAs, the major iso‐branched (ibr) and anteiso‐branched (aibr) chain FFAs had a chain length between 14 and 17 carbon atoms. Sebum from the LS skin type was polarized towards the ibr FA 13Me‐C15:0 and the aibr FA 14Me‐C17:0, whereas sebum from the HS group presented prevalently ibr FFAs with a carbon number between 14 and 16 (Figure 1B). C16:1 and C18:1 were the most abundant MUFAs (Figure 1C). Except for 13Me‐C15:0, C19:0, C21:0, and the polyunsaturated FA (PUFA) C20:2, all FFAs were more abundant in HS subjects (Table S1A). By contrast, FAOHs were equally abundant in the two groups.

The weight/weight percentage (%) of the major lipid classes was evaluated at the OP in sebum, whose total amounts were obtained by summing the individual lipids within each class. The weight/wt% showed the characteristic distribution of the sebaceous secretion, with FFAs and TGs accounting together for about 60% of the total mass [15]. Nevertheless, while the weight/wt% of FFAs and squalene was higher in the HS skin type, that of TGs and cholesterol was significantly higher in the LS group (Table S1B).

The HS to LS ratios of the sebum lipid abundance reported in the Table S1A were ordered according to their decreasing values to obtain a ranking of the differences observed between the two skin types (Figure S2). Red bars represent lipid compounds with a fold change (FC) ≥ 2. Some branched FAs, MUFAs, and odd‐chain FAs presented abundance more than 4‐fold higher in the HS group. FA desaturase (FADS2) and stearoyl CoA desaturase (SCD1) are characterized for their site‐specific insertion of a C‐C double bond [15]. Sapienate (C16:1n‐10) is the sebum‐specific MUFA produced by the FADS2 activity expressed in differentiating sebocytes in the suprabasal layer of the SG [40]. FADS2 also catalyzes the desaturation of the odd chained FA C17:0 [41, 42]. Isomers of the C18:1 FA are less characterized in human sebum. LS skin presented a different distribution of MUFAs, being C16:1 sensibly depleted compared to C18:1 in the LS skin type (Table S1A). The index of FADS2 enzymatic activity given by the C16:1/C16:0 ratio was more than 2‐fold higher in HS skin, followed by the C18:1/C18:0 and the C17:1/C17:0 ratios.

Lipid synthesis is a highly orchestrated process. Lipids convey structural functions and act as signaling molecules in several physiological and pathological conditions [43]. Apart from protective functions, sebum lipids may play an active role in modulating lipid pathways in the epidermal compartment [7]. A clear pattern of associations emerged in the lipid to lipid and the lipid to enzymatic pathways comparisons (Figure S3). The interrelationship between sebum lipid abundances was apparent in both skin groups. Nevertheless, associations were stronger and more extensive in the HS group. Squalene, cholesterol, glycerolipids (TGs, and DGs), and WEs were positively correlated with FFAs in HS skin type. Squalene correlates positively with other complex sebum lipids, i.e., glycerolipids and WEs, at the cheeks, at both OP and ELP. Direct correlation between squalene and vitamin E was found in both LS and HS groups, at both facial sites during ELP, and in sebum from cheeks in the OP. The sebum secretion is a physiologic pathway for the delivery of vitamin E onto the skin surface. Co‐secretion of vitamin E and squalene is functional to the antioxidant defense system on the skin [44, 45]. The desaturation indexes expressed as the ratio of MUFA to SFA of the same chain length (C16:1/C16:0, C17:1/C17:0, and C18:1/C18:0) were positively correlated with all kinds of FFAs in both LS and HS skin, regardless of the OP or the ELP menstrual phases. This association was independent of the abundance of sebum, as the MUFA/SFA ratio was a non‐dimensional parameter.

### 3.2 Profiles of Sebum Lipids Distribution in Two Facial Sites at the OP and ELP in HS and LS Skin Types

Table 1 reports μg/tape (average ± SD) of each sebum component and *p*‐values of differences between foreheads and cheeks in two major phases of the menstrual cycle, i.e., OP and ELP. Overall sebum composition of foreheads was consistent with that observed in cheeks in both skin types, as seen in weight/wt% of different lipid classes, except for individual lipids that presented different patterns of abundance in foreheads and cheeks during the two menstrual phases. In general, differences in the sebum composition between foreheads and cheeks were more apparent during the ELP phase. In the LS group, stearic acid (C18:0) was significantly lower on the forehead, regardless of the phase. Laurate (C12:0) and myristate (C14:0) were higher on cheeks at the ELP, together with cholesterol. The HS group was characterized by higher levels of branched FFAs on the cheeks at the ELP. Interestingly, TGs were higher on foreheads, regardless of the skin type and the hormonal phase (Table 1). C14:1 was higher on foreheads in HS subjects at the OP. The overall amounts of sebum, obtained by summing the quantified μg/tape of the single species, showed even distribution between cheeks and foreheads in both skin types. Interestingly, the weight/wt% of pooled lipid classes was consistent between LS and HS types in the OP and ELP, except for cholesterol and FFAs that switched their relative abundance in the LS and HS groups, respectively. In general, cheeks displayed a higher percentage of FFAs and lower relative abundance of squalene and TGs as expected by the higher density of the SGs at the forehead in comparison with the cheeks.

**TABLE 1:** Average μg/tape and standard deviation of sebum lipids in forehead and cheek sites in both low sebometry (LS) and high sebometry (HS) skin groups evaluated at the OP and ELP. Darker blue and red indicated the highest value of each lipid in either forehead or cheek sites, in the LS and HS groups, respectively. Significance of the difference between the two sites were indicated with *p*‐values.

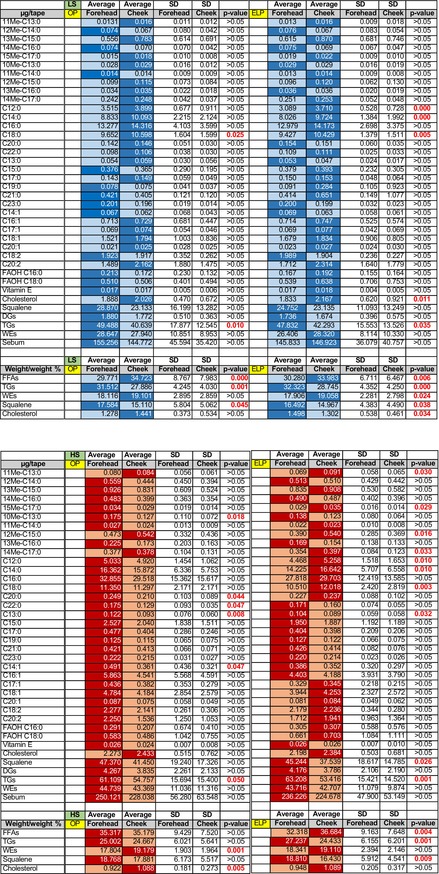

*Note:* Data are expressed as average ± SD of the lipid amounts (μg/tape) and vitamin E (ng/tape) in sebum from forehead and cheek areas in the ovulatory and early luteal phases, indicated with OP and ELP, respectively. ELP, early luteal phase; OP, ovulation phase; SD, standard deviation.

### 3.3 Changes of the Sebum Lipid Profiles During the Four Main Phases of the Menstrual Cycle

**FIGURE 2:**
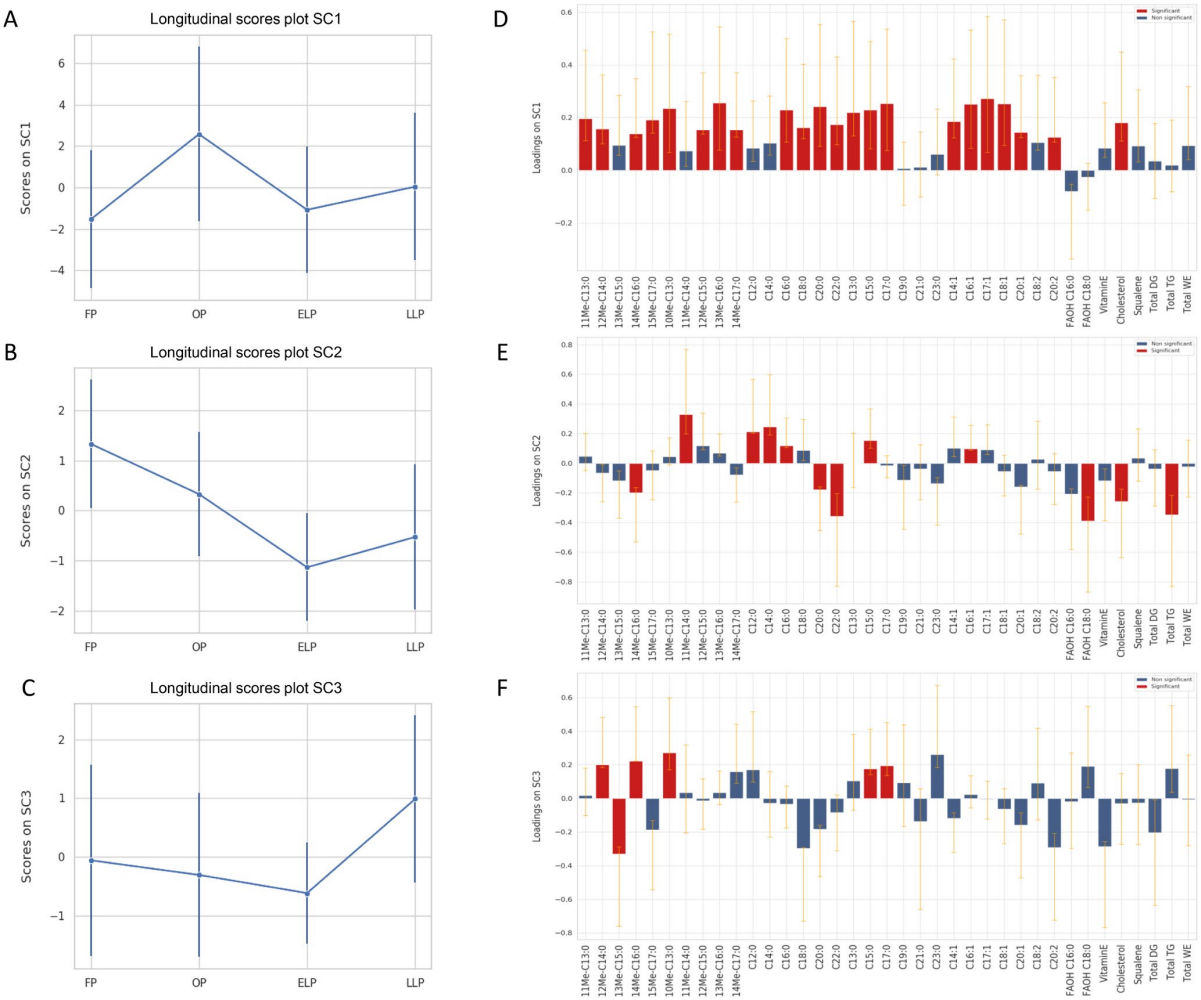
ANOVA‐simultaneous component analysis (ASCA) analysis on the effect matrix for hormone phases on foreheads sebum components in high sebometry (HS) skin. Scores plot after projection of the residuals onto the space spanned by the three simultaneous components: SC1 (A), SC2 (B), and SC3 (C). To highlight the longitudinal trend, for each component, mean scores are displayed as a function of the hormone phase and the confidence intervals are calculated based on the back‐projected residuals. Variable loadings on SC1 (D), on SC2 (E), and on SC3 (F), together with their confidence interval (red and blue bars indicate significantly and not significantly contributing descriptors, respectively). By comparing the positive or negative signs of the loadings with those of the different hormone phases in the related SC score plot, it could be defined how the lipid species varied along the menstrual cycle. Red and blue bars indicated lipids that varied significantly and not significantly, respectively.

In the overall data classified according to three factors, i.e., skin type, face site, and menstrual phase, the significant effects were due to the skin type, with dominant sebum lipids in the HS group. Moreover, looking at the data in the two face sites, and to the skin type/facial site interaction, it appeared that the amounts of lipid metabolites changed more clearly when foreheads were examined for their sebum profiles during OP and ELP in the HS group (see Table 1). This observation agrees with the literature data [5]. Thus, the changes in the lipid profiles were examined by ASCA applied to the foreheads data in the HS subgroup across the principal four phases of the menstrual cycle, after centering the data on each panelist (Rem‐ANOVA). The multivariate analysis allowed associating the covariation of lipid metabolites in sebum coincidentally with a specific menstrual phase [46, 47, 48]. The histogram in the Figure S4 shows that the effect due to the menstrual phases was significant, as indicated by the *p*‐value (*p* = 0.003) marked by the red line positioned away from the histogram. The scores along the three simultaneous components (SCs) SC1, SC2, and SC3, which were associated with the factor ‘menstrual phases’, were represented in the respective longitudinal view in panels A, B, and C in Figure 2. The summary of variations of sebaceous lipids resulted in being significant across the menstrual phases. To interpret the score trends, the loadings were investigated according to their values represented in the loadings plots of panel D, E, and F in Figure 2. Apparent changes in the sebum lipid abundance were likely to account for the effects that the hormones exerted on the SG activity, provided that the FP is coincident with the start of ascending levels of estradiol (E2), which then peaks shortly before the OP, together with the follicle stimulating hormone (FSH) and luteinizing hormone (LH). Progesterone levels rise in the ELP. In contrast, both the initial and the terminal FP and LLP, respectively, are characterized by the decline in both E2 and progesterone [31]. At the OP, most branched and straight chain FFAs were significantly elevated in sebum together with MUFAs and cholesterol (Figure 2A,D). The ibr FA 14Me‐C16:0 and cholesterol were still high in the ELP, while the aibr FA 11Me‐C14:0, and the straight SFAs C12:0, C14:0, C15:0, and C16:0 and the MUFA C16:1 decreased at the ELP. In this phase, longer straight SFAs, such as C20:0 and C22:0 together with the FAOH C18:0 and TGs were higher (Figure 2B,E). In the final stage, i.e., the LLP, the 12Me‐C14:0 started to increase together with 14Me‐C16:0, 10Me‐C13:0, C15:0, and C17:0 FFAs, whereas the ibrFA 13Me‐C15:0 presented lower levels (Figure 2C,F). Overall, two major patterns of variations were observed in the centered lipid amounts. Sebum‐type lipids, which included branched FFAs, C15‐18 long SFAs, and C16‐18 long MUFAs, tended to peak at OP and to decline at the ELP, as shown in the Figure S5A,B. Epidermal‐type lipids, such as long chain SFAs C20:0 and C22:0, together with cholesterol, presented higher amounts at OP that were maintained along the following phases (Figure S5C).

To study the impact of E2 and progesterone on the regulation of genes involved in cholesterol homeostasis, de novo FA synthesis, and FA desaturation, we used the human SG cells SZ95 [49], which express estrogen receptors [50, 51]. Supporting Information and methods of the cell experiments are reported in Appendix S3. At 48 and 72 h, both E2 and progesterone (P) significantly increased the mRNA expression level of Sterol Regulatory Element‐Binding Protein 1 (SREBP‐1), which plays a crucial role in regulating lipid and cholesterol metabolism by activating the transcription of genes involved in FA and cholesterol synthesis. Combined treatment with E2 and P did not show any additive effect. P at 48 h and E2 at 72 h significantly induced the mRNA levels of FA synthase (FASN), which plays a crucial role in de novo FA synthesis. FADS2 transcript levels were significantly increased only in SZ95 sebocytes treated with the E2 + P combination for 72 h, whereas SCD1 was induced only by E2 after 72 h. Neither E2 nor P influenced the expression of peroxisome proliferator‐activated receptor gamma (PPARγ), another key player in lipid metabolism (Figure S6A). We then evaluated the lipid content of SZ95 sebocytes by Nile Red assay after treatment with E2, P, or their combination (Figure S6B). The E2 + P combination induced a slight decrease in neutral lipid content after 48 h of treatment. Both hormones resulted in significantly lower polar lipid content at 48 and 96 h; nevertheless, the neutral to polar lipid balance was in favor of the neutral component.

## 4 Discussion

The SG is an endocrine oil‐producing appendage harnessed with enzymes of lipid metabolism and receptors for diverse hormones [23]. Due to the high density of SG on the face, it is likely that fluctuations in female hormone levels may result in skin perturbation in this SGR area. Forehead and cheeks account for facial sites with high and low levels of sebum secretion, respectively [37, 38, 39]. Whether or not the distribution of sebum lipids matches the distribution of hormone receptors on foreheads and cheeks awaits clarification. The amount of sebum secreted is likely to be related to the menstrual cycle [30]. Nonetheless, direct evidence of the effect of female hormone on sebum production is lacking. The SG expresses both ERα and ERβ receptors and, in the nucleolus, the progesterone receptors [24]. The effects of estrogens have been investigated on human skin and SG, while those of endogenous progesterone are less explored. In earlier studies, the lowest SER have been observed at the peak of concentration of the circulating E2 [52]. Levels of estrogens decline together with those of progesterone and remain low coincidentally with bleeding and the early FP [26]. Changes in the sebum composition of skin occurred coincidentally with peaking of female hormones in humans. Before and during the bleeding, there is a trend towards decreased hydration and poor barrier [27]. Skin tends to be drier in the first week, whereas sebum secretion appears to be high between the second and third week of the hormonal cycle [27] in line with our findings of elevation of certain sebum‐type lipids at the ovulation. Thus, poor barrier and the decline of the E2 levels characteristic of the perimenstrual phase are likely related. Variable effects of progesterone on sebum production have been observed in animals, depending on the species and the sex [25]. In our study, we compared areas of the T‐zone (forehead) and the U‐zone (cheeks) in two major skin types, HS and LS, at time points coincident with the E2 peak and valley [52, 53]. Detectable differences were observed between forehead and cheek in both LS and HS skin types and along the menstrual phases corresponding to major changes in estrogens, progesterone, FSH, and LH. While differences between the two facial zones were detectable in LS skin at both the OP and ELP, changes during the menstrual phases were more apparent in HS skin, especially at the ELP phase. The distribution of SFAs was consistently represented in the LS and HS skin types; yet, the absolute amounts of each SFA and their overall abundance were clearly higher in the HS group. In particular, here, palmitate (C16:0) was 2‐fold more abundant. Especially the profiles of abundance of branched FFAs and MUFAs were visibly different in the two skin types. The HS group had more than 2‐fold higher levels of the branched FFA members and of sapienate (C16:1n‐10), which is formed by insertion of a double bond at the Δ6 position by the FADS2 enzyme [42]. This result indicates that sebum‐specific pathways are constitutively more active in HS. In human skin, mRNA and protein expression of FADS2 is apparent in the suprabasal layers of the SG, enriched in differentiating sebocytes [40]. More active SGs secrete a higher proportion of the sebaceous C16:1n‐10 [22]. Analogously, the occurrence of branched FFAs in sebum can be linked to inherent sebaceous biosynthetic pathways and microbial metabolism [54, 55, 56, 57]. Branched short‐chain organic acids and amino acids are the candidate precursors of the branched FAs [58]. LS and HS groups presented skin type‐specific distribution of SFAs between forehead and cheek. In contrast, TG levels were higher on the foreheads, regardless of the skin type. Notably, differences in the androgen receptor expression at the T and U zones may contribute to the differences in sebum production in the two skin types [19]. C16:1 has been reported to be directly correlated with urine testosterone levels in both males and females [59]. The SG is characterized by a high FA *de novo* synthesis catalyzed by the FA synthase [60]. Biosynthesis of FFAs and squalene together is pivotal in the maturation of sebocytes and sebum production. Once secreted, sebum offers substrates for microbial colonization, which, in turn, partly transforms the native lipids, mainly by hydrolysis of TGs, a process yielding FFAs. An early study reported that the levels of both cholesterol and TGs, the latter making up the bulk of sebum mass, increase during the menstrual cycle [61]. Cholesterol, which is referred to as an epidermal‐type lipid, raises during the OP and ELP, suggesting that the epidermal compartment may participate in the response to menstrual hormones [62]. In turn, the epidermal lipids present a different profile of abundance in SGR areas [7]. Dysfunctions of the epidermal barrier contribute to the exacerbation of acne manifestations, which worsen perimenstrually [63]. Nevertheless, the implications of the female hormones in the SG metabolism are also to be placed in the context of systemic metabolic effects. In a study investigating the molecular phenotype of menstrual cycle phases associated with female hormone rhythmicity, it was shown that the plasma metabolome changed significantly between phases. In particular, the anabolic shift was coincident with the luteal phase, likely due to increased utilization of fat for lipid and steroid synthesis [31]. In SZ95 sebocytes, E2 and progesterone, while imparting suppression of lipidogenesis, activate the transcription of lipogenic genes at advanced stages, consistent with the fluctuating levels of SSL during the menstrual phases. The results of this study support the translation of hormonal fluctuations into specific fingerprints of sebaceous lipids. The overall data suggest that female hormones regulating the menstrual cycle impact pathways of sebogenesis, including FA desaturation and de novo FA synthesis from both canonical and non‐canonical FA precursors, as well as cholesterol turnover and squalene accumulation in the SG, confirming previous clinical studies [30].

## Conflicts of Interest

E.C. was the principal investigator at the host institution funded by Procter and Gamble; A.C., G.B., and M.M. were supported with a fellowship under the research project funded by Procter and Gamble; H.F.L., V.P., Y.X., and H.Z. are employees of The Procter and Gamble Company, which covered the entire costs of this work. The authors have no financial interests in the manuscript.

## Supporting information


**Figure S1:** Schematization of the menstrual phases and sebum sampling applied to the regular menstruation cycle of 28 days.

**Figure S2:** Rank of the high sebometry (HS)‐low sebometry (LS) group ratio in the sebum lipid amounts. Ratio of amounts of individual components between HS skin and LS skin women quantified in foreheads and cheeks in correspondence of the ovulation phase of the menstrual cycle. HS to LS skin ratio were reported for each quantified lipid.

**Figure S3:** Heatmaps of Spearman's correlations among sebum components, total sebum amount, and indexes of monounsaturation (C16:1/C16:0, C17:1/C17:0, and C18:1/C18:0) determined on cheeks and foreheads of low sebometry (LS) and high sebometry (HS) skin groups at the ovulation phase (OP), and the early luteal phase (ELP). Color scale of correlations is provided for each panel. Shades of red and blue colors indicated direct and inverse correlation, respectively. Asterisks indicate significant correlation (*p < 0.05; **p < 0.005).

**Figure S4:** ASCA model on absolute data from foreheads. Sum of squares of the effect matrix (red line) compared to the corresponding distribution under the null hypothesis estimated by permutation tests (blue histograms).

**Figure S5:** Box plots of sebum‐type branched and odd‐chain FFAs (A); SFAs and MUFAs (B); epidermal type long‐chain FFAs and cholesterol (C). Data were analyzed using MatLab (version 8.6.0 release R2015b; The Mathworks, Natick, MA) and Python custom scripts that leveraged the scikit‐learn library. Continuous variables were represented as median values with confidence intervals or mean ± standard deviation (SD). Kruskal‐Wallis test was used to assess significance among the FP, OP, ELP, and LLP phases. Data represent the centred amounts (μg) of lipid species. Differences were considered statistically significant with *p* ≤ 0.05 (*p < 0.05, **p < 0.005).

**Figure S6:** (A) Quantitative real time PCR analysis of lipidogenic genes in SZ95 sebocytes treated with vehicle, or 1 μM 17β‐estradiol (E2), 1 μM progesterone (P), or the combination of both sex hormones for 24–48–72 h. All mRNA values were normalized against the expression of GAPDH and were reported relative to control (vehicle). Data represent the mean ± SD of three independent experiments (*p < 0.05 vs. vehicle); (B) Determination of neutral lipids, polar lipids and neutral‐to‐polar lipid ratio by Nile Red assay in SZ95 sebocytes treated with vehicle, or 1 μM E2, 1 μM P, or the combination of both sex hormones for 48–96 h. Data represent the mean ± SD of three independent experiments in hexaplicate. The values were expressed as relative to the control (vehicle, set as 1) (*p < 0.05, **p < 0.01 vs. vehicle).

**Table S1:** Pooled quantitative data of lipids from both sites and the OP and ELP. (A) Averaged μg/tape for each lipid, except vitamin E expressed as ng/tap, represented in the ranking of the oily‐to‐dry ratio in Figure S2; (B) Weight/weight percentage (%) of the major lipid classes.

**Appendix S1:** List of target sebum lipids with technical and analytical details.

**Appendix S2:** Dominant influencing factors considered for the inclusion/exclusion of the study participants.

**Appendix S3:** Description of ANOVA‐simultaneous component analysis (ASCA) and Supporting Information and methods; Table S1: Quantitative results of sebum components in foreheads and cheeks.

## Data Availability

The data that support the findings of this study are available on request from the corresponding author. The data are not publicly available due to privacy or ethical restrictions.
