# Supplementary figures and images for: Physiological Differences in Sebum Composition in Regularly Menstruating Healthy Women
Source: J Dermatol. 2025 Aug 28;52(11):1638–47. doi: 10.1111/1346-8138.17908 (PMC12592595; doi:10.1111/1346-8138.17908)

Supplementary Figure S1

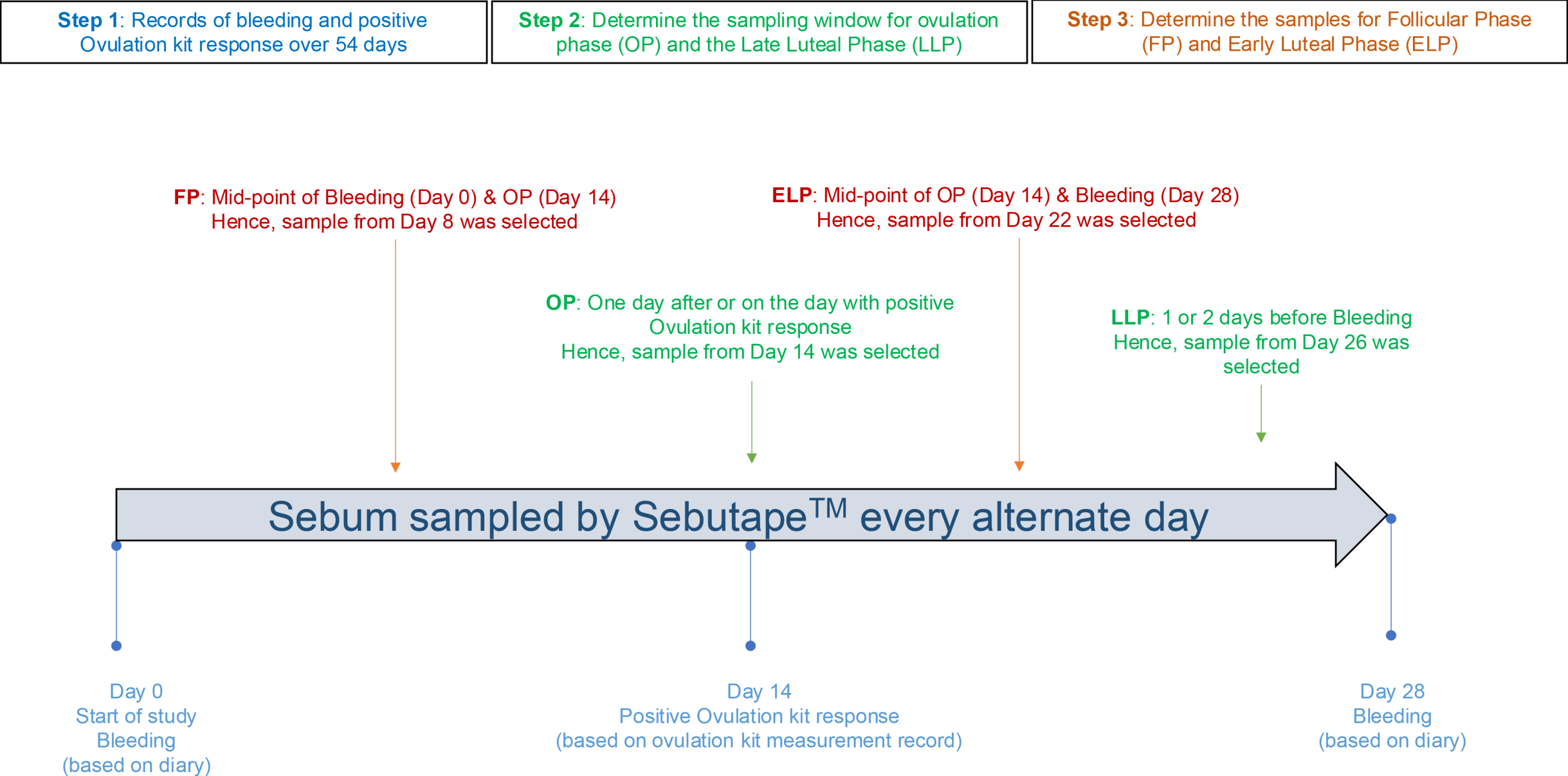

Supplement: Supplementary file 1 — Figure S1: Schematization of the menstrual phases and sebum sampling applied to the regular menstruation cycle of 28 days. [file JDE-52-1638-s003.pdf]

## Supplementary Figure S2

### High Sebometry Group/ Low Sebometry Group Ratio

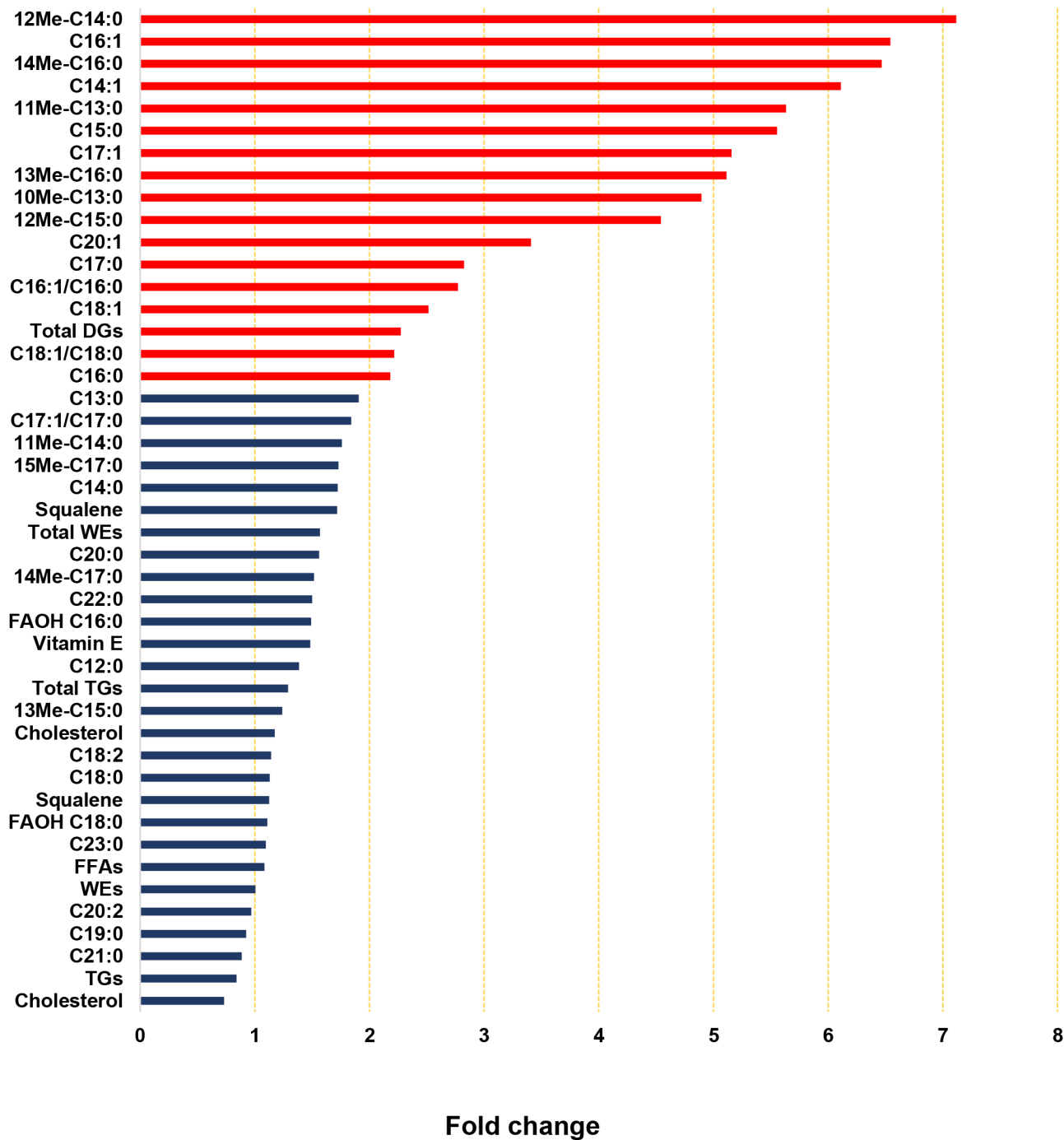

Supplement: Supplementary file 2 — Figure S2: Rank of the high sebometry (HS)‐low sebometry (LS) group ratio in the sebum lipid amounts. Ratio of amounts of individual components between HS skin and LS skin women quantified in foreheads and cheeks in correspondence of the ovulation phase of the menstrual cycle. HS to LS skin ratio were reported for each quantified lipid. [file JDE-52-1638-s010.pdf]

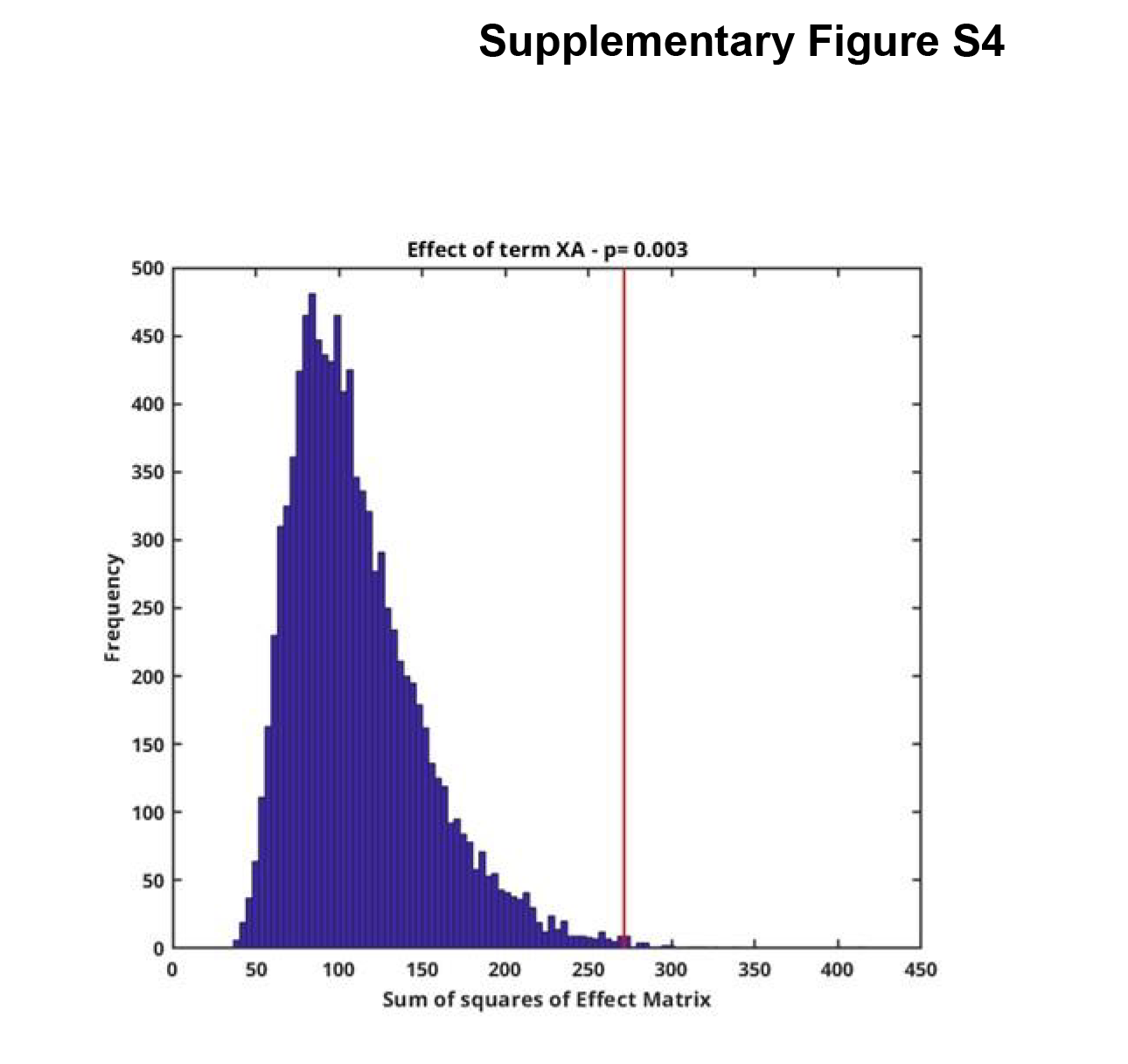

Supplement: Supplementary file 4 — Figure S4: ASCA model on absolute data from foreheads. Sum of squares of the effect matrix (red line) compared to the corresponding distribution under the null hypothesis estimated by permutation tests (blue histograms). [file JDE-52-1638-s009.tif]

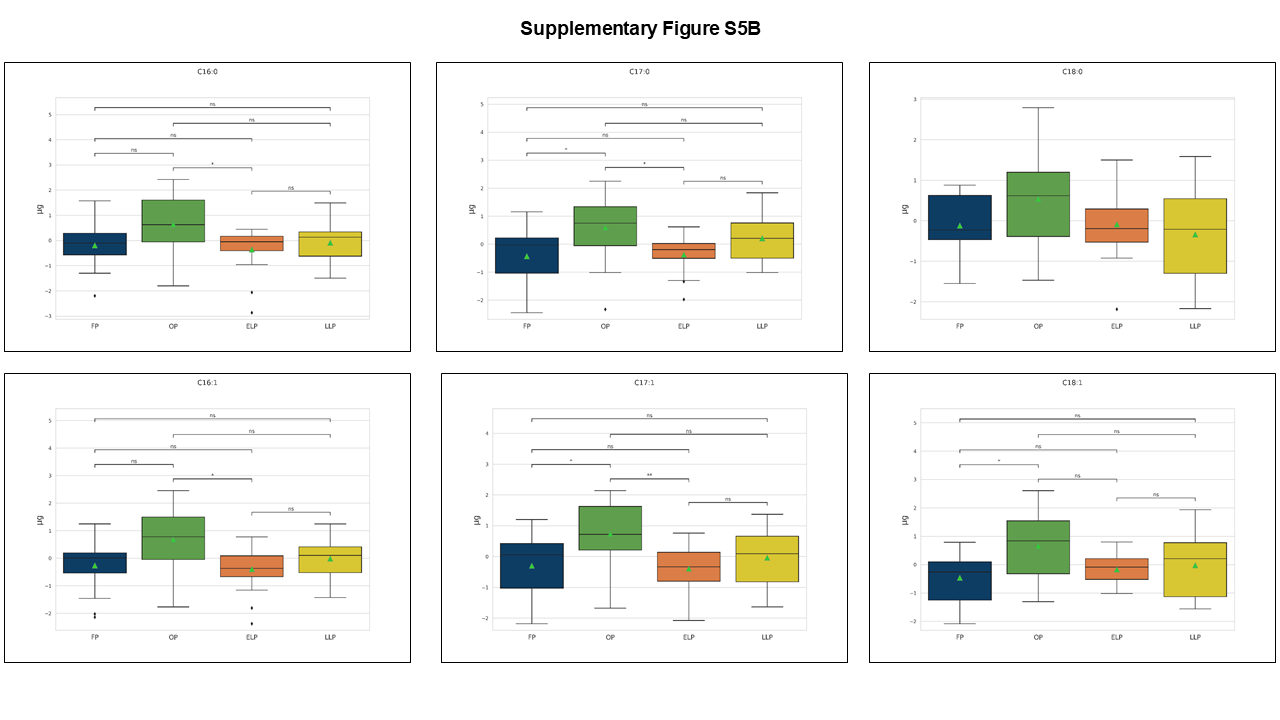

Supplement: Supplementary file 5 — Figure S5: Box plots of sebum‐type branched and odd‐chain FFAs (A); SFAs and MUFAs (B); epidermal type long‐chain FFAs and cholesterol (C). Data were analyzed using MatLab (version 8.6.0 release R2015b; The Mathworks, Natick, MA) and Python custom scripts that leveraged the scikit‐learn library. Continuous variables were represented as median values with confidence intervals or mean ± standard deviation (SD). Kruskal‐Wallis test was used to assess significance among the FP, OP, ELP, and LLP phases. Data represent the centred amounts (μg) of lipid species. Differences were considered statistically significant with p ≤ 0.05 (*p < 0.05, **p < 0.005). [file JDE-52-1638-s006.zip › Supplementary S5B R1 24-7-25.tif]

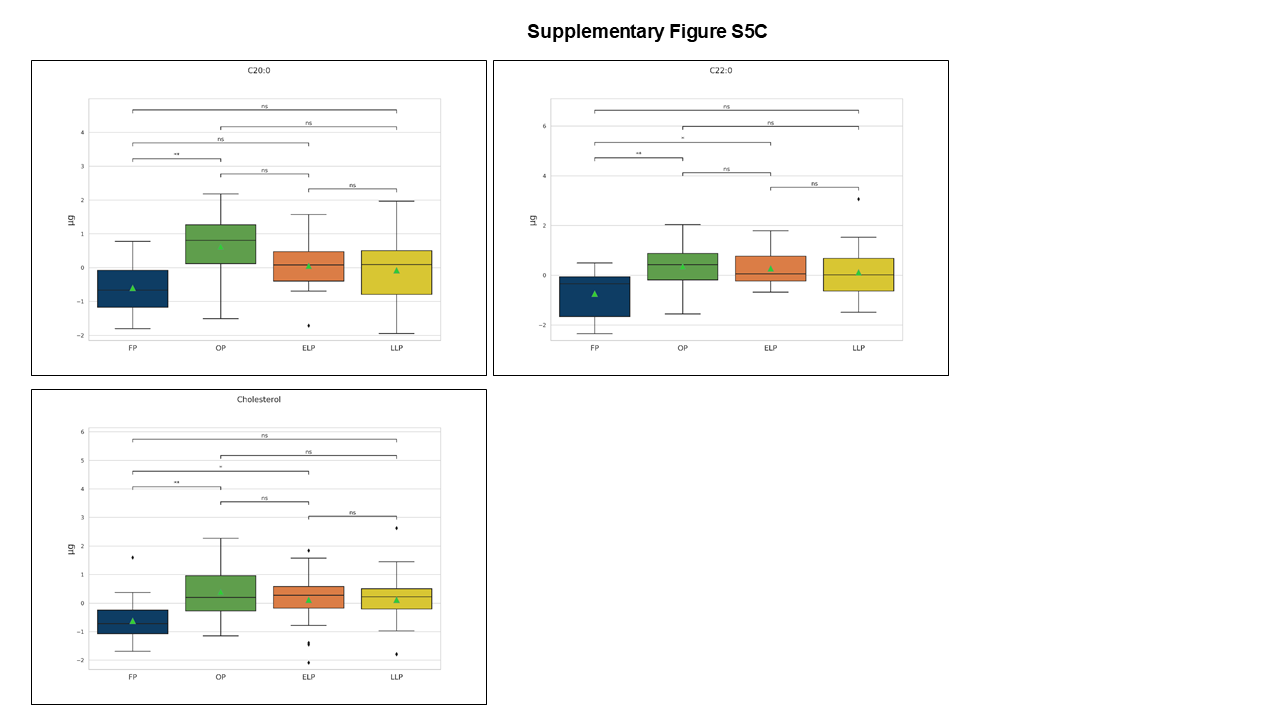

Supplement: Supplementary file 5 — Figure S5: Box plots of sebum‐type branched and odd‐chain FFAs (A); SFAs and MUFAs (B); epidermal type long‐chain FFAs and cholesterol (C). Data were analyzed using MatLab (version 8.6.0 release R2015b; The Mathworks, Natick, MA) and Python custom scripts that leveraged the scikit‐learn library. Continuous variables were represented as median values with confidence intervals or mean ± standard deviation (SD). Kruskal‐Wallis test was used to assess significance among the FP, OP, ELP, and LLP phases. Data represent the centred amounts (μg) of lipid species. Differences were considered statistically significant with p ≤ 0.05 (*p < 0.05, **p < 0.005). [file JDE-52-1638-s006.zip › Supplementary S5C R1 24-7-25.tif]

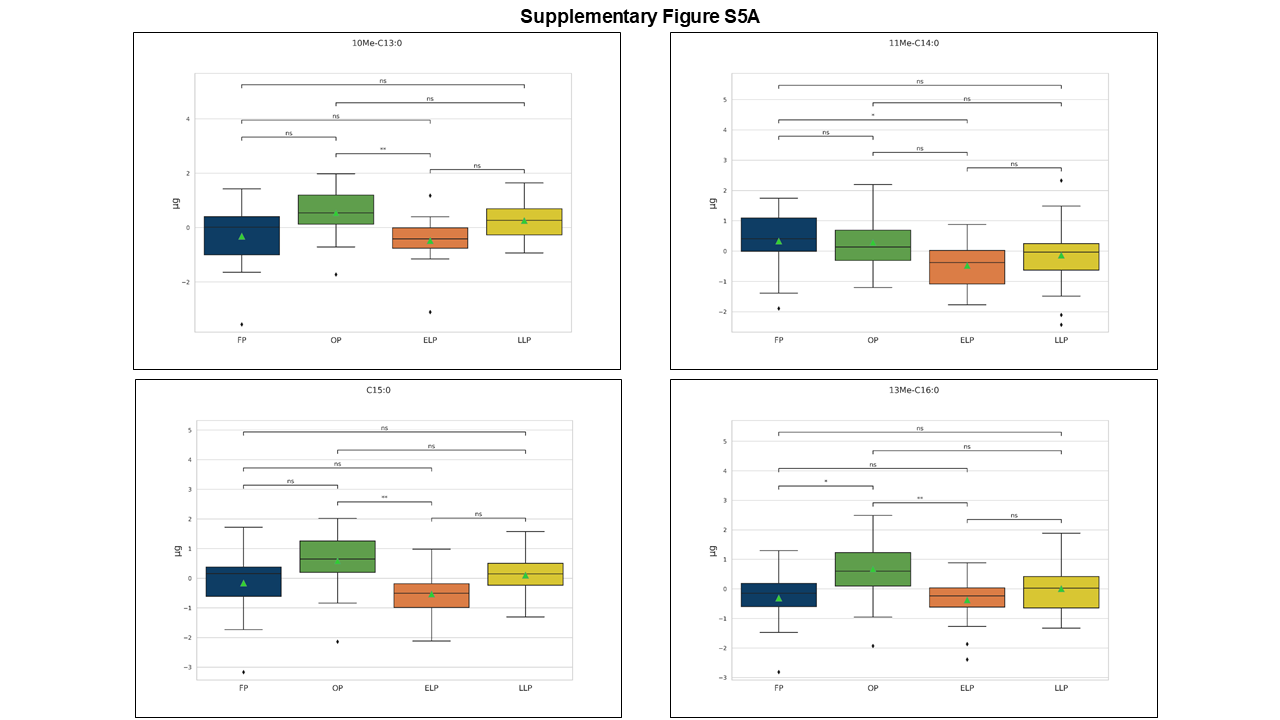

Supplement: Supplementary file 5 — Figure S5: Box plots of sebum‐type branched and odd‐chain FFAs (A); SFAs and MUFAs (B); epidermal type long‐chain FFAs and cholesterol (C). Data were analyzed using MatLab (version 8.6.0 release R2015b; The Mathworks, Natick, MA) and Python custom scripts that leveraged the scikit‐learn library. Continuous variables were represented as median values with confidence intervals or mean ± standard deviation (SD). Kruskal‐Wallis test was used to assess significance among the FP, OP, ELP, and LLP phases. Data represent the centred amounts (μg) of lipid species. Differences were considered statistically significant with p ≤ 0.05 (*p < 0.05, **p < 0.005). [file JDE-52-1638-s006.zip › Supplementary S5A R1 24-7-25.tif]

# Supplementary Figure S6

**A**

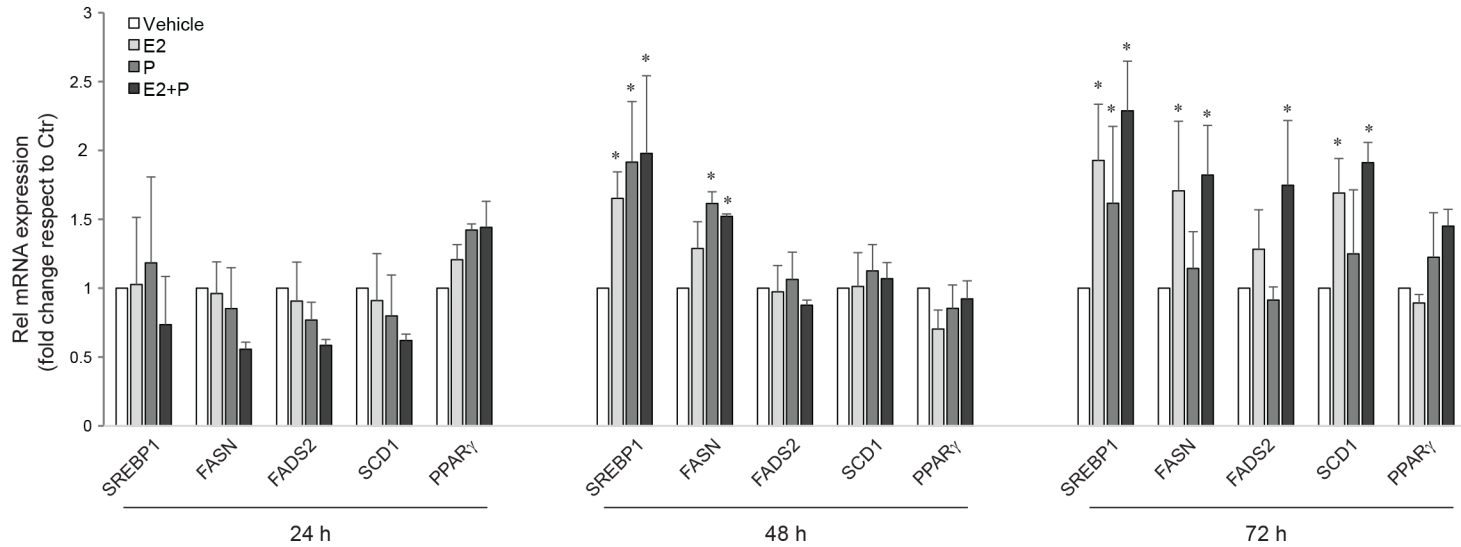

**B**

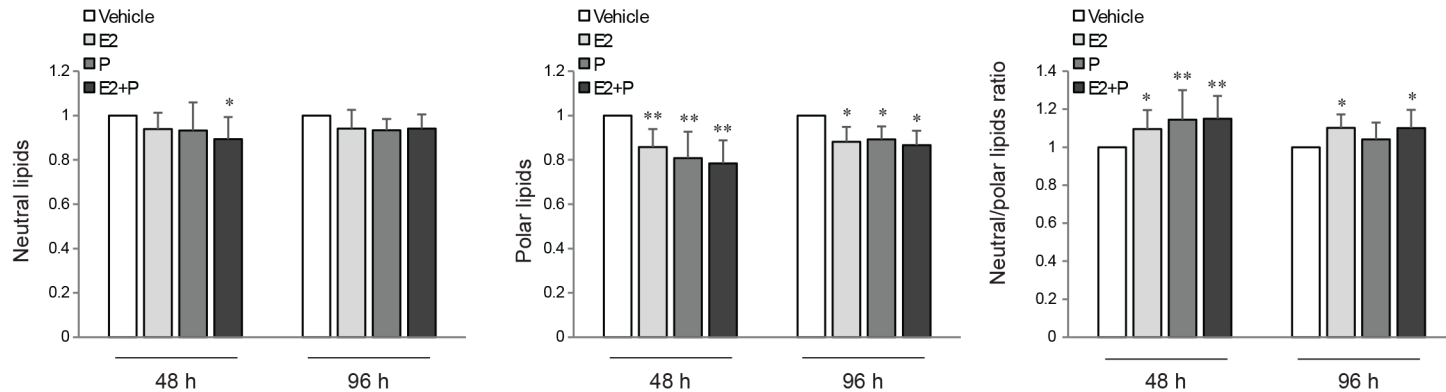

Supplement: Supplementary file 6 — Figure S6: (A) Quantitative real time PCR analysis of lipidogenic genes in SZ95 sebocytes treated with vehicle, or 1 μM 17β‐estradiol (E2), 1 μM progesterone (P), or the combination of both sex hormones for 24–48–72 h. All mRNA values were normalized against the expression of GAPDH and were reported relative to control (vehicle). Data represent the mean ± SD of three independent experiments (*p < 0.05 vs. vehicle); (B) Determination of neutral lipids, polar lipids and neutral‐to‐polar lipid ratio by Nile Red assay in SZ95 sebocytes treated with vehicle, or 1 μM E2, 1 μM P, or the combination of both sex hormones for 48–96 h. Data represent the mean ± SD of three independent experiments in hexaplicate. The values were expressed as relative to the control (vehicle, set as 1) (*p < 0.05, **p < 0.01 vs. vehicle). [file JDE-52-1638-s008.pdf]
