# Supplementary material for: Physiological Differences in Sebum Composition in Regularly Menstruating Healthy Women
Source: J Dermatol. 2025 Aug 28;52(11):1638–47. doi: 10.1111/1346-8138.17908 (PMC12592595; doi:10.1111/1346-8138.17908)

Supplementary Figure S3

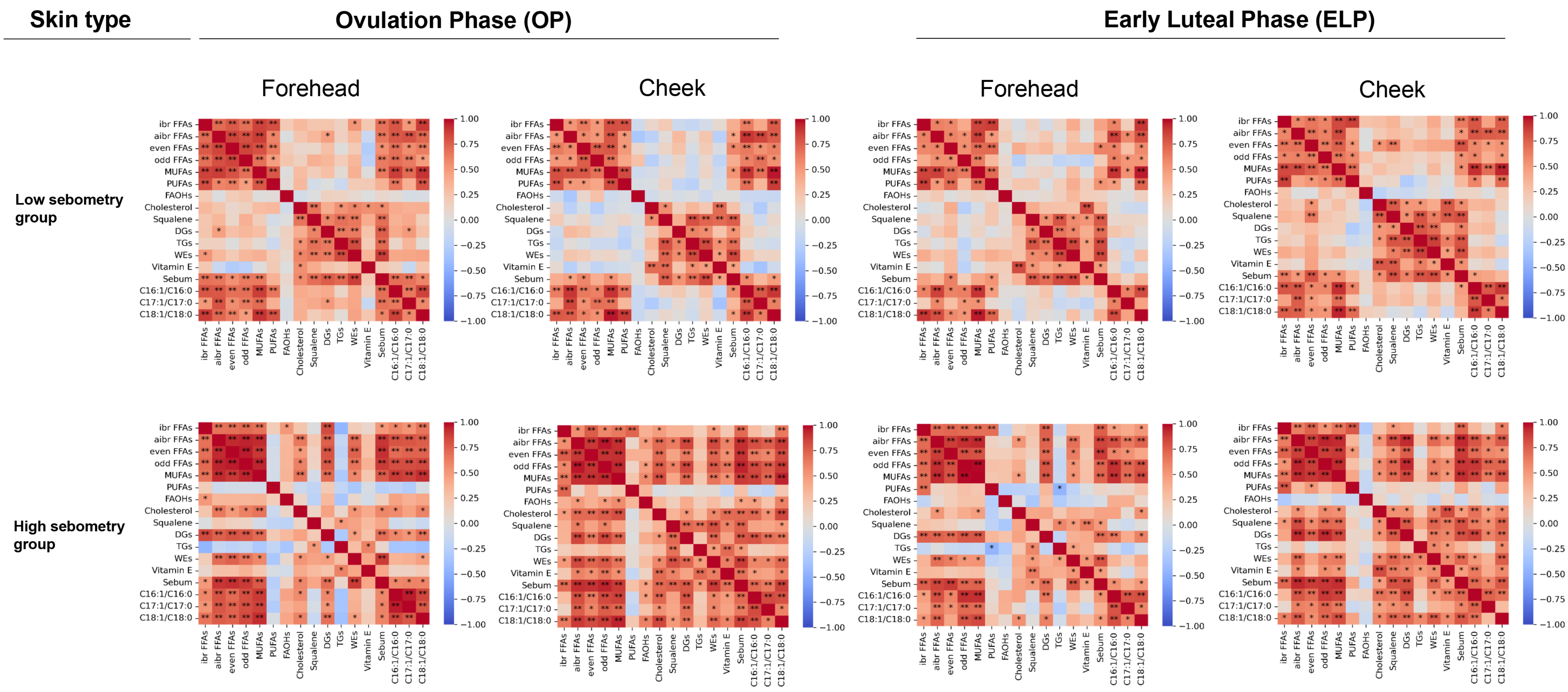

Supplement: Supplementary file 3 — Figure S3: Heatmaps of Spearman's correlations among sebum components, total sebum amount, and indexes of monounsaturation (C16:1/C16:0, C17:1/C17:0, and C18:1/C18:0) determined on cheeks and foreheads of low sebometry (LS) and high sebometry (HS) skin groups at the ovulation phase (OP), and the early luteal phase (ELP). Color scale of correlations is provided for each panel. Shades of red and blue colors indicated direct and inverse correlation, respectively. Asterisks indicate significant correlation (*p < 0.05; **p < 0.005). [file JDE-52-1638-s007.pdf]
