# Supplementary material for: Physiological Differences in Sebum Composition in Regularly Menstruating Healthy Women
Source: J Dermatol. 2025 Aug 28;52(11):1638–47. doi: 10.1111/1346-8138.17908 (PMC12592595; doi:10.1111/1346-8138.17908)
Supplement: Supplementary file 9 — Appendix S2: Dominant influencing factors considered for the inclusion/exclusion of the study participants. [file JDE-52-1638-s005.docx]

**Appendix 2**

Dominant influencing factors considered for the inclusion/exclusion of the study participants.

**Key inclusion criteria**

- Healthy Chinese female, age 24-29
- Lived in Xi’an in past 10 years
- Had regular menstrual cycle, can tell exact date of bleeding, and duration of full cycle between 26-30 day.
- Self-assessed as non-oily skin both T zone and U zone.
- Overnight >12hr sebum casual level (middle forehead) ≤70 by Sebumeter® reading.
- Self-assessed as oily skin both T zone and U zone.
- Overnight >12 hours sebum casual level (middle forehead) ≥150 by Sebumeter® reading.

**Key exclusion criteria:**

- Self-assessed as normal skin or combination skin
- Smoking in past 5 years
- Obvious facial acne in past year, or noticeable facial acne (both inflammatory and none-inflammatory) in past menstrual cycle
- Severe fine line, wrinkle, texture, pore and facial acne scar (clinically assessed)
- Pregnancy, nursing, or planning pregnancy
- Intensive outdoor activities
- Oral vitamin supplements in the past 6 months
- Use of products containing niacinamide in past 6 months
- History of heart disease, musculoskeletal, chronic obstructive pulmonary disease COPD
- Turner Syndrome (inherited condition, underdeveloped sex characteristics)
- Hypopituitarism
- Hyperthyroidism
- Hypertension or increase blood pressure recently
- Anemia
- Taking glucocorticosteroid; ampicillin, or phenothiazine
- Taking psychotropic drugs (e.g., dopamine inhibitors).
- Alcohol and coffee consumption.
