# Supplementary material for: Physiological Differences in Sebum Composition in Regularly Menstruating Healthy Women
Source: J Dermatol. 2025 Aug 28;52(11):1638–47. doi: 10.1111/1346-8138.17908 (PMC12592595; doi:10.1111/1346-8138.17908)
Supplement: Supplementary file 10 — Appendix S3: Description of ANOVA‐simultaneous component analysis (ASCA) and Supporting Information and methods; Table S1: Quantitative results of sebum components in foreheads and cheeks. [file JDE-52-1638-s002.docx]

**Appendix 3**

**ANOVA-simultaneous component analysis (ASCA)**

Due to the advantage of ASCA of coupling multivariate ANOVA decomposition of the experimental data matrix with non-parametric testing and interpretation of the multivariate effects through the use of principal component analysis (PCA) it was applied to the data matrices on sebum. In detail, by indicating as **X** the matrix collecting the results of the designed experiments, the first step of the procedure is to partition the variability in it, according to the ANOVA scheme, i.e., as a sum of additive terms, each accounting for the effect of a particular design term (factor or interaction). In the case where a single factor is of interest, such as in the present study, the data matrix is decomposed as follows:

**X**=**X**_m_+**X**_phase_+**X**_res_

All these matrices have the same dimensions, but are constructed in different ways. In **X**_m_, each row contains the mean experimental profile calculated on all the samples; indeed, this “grand mean” matrix is introduced just to express the variation induced by the factor(s) as differences with respect to the mean profile. **X**_phase_ is the matrix accounting for the effect of hormonal phase. Since the factor has four levels levels (or time points), FP, OP, ELP, and LLP, the matrix is built accordingly: All the rows of **X**_phase_ corresponding to samples at the OP phase will contain identical copies of the mean profile recorded at this time point, and the same occurs for the other phases. Lastly, **X**_res_ is the residual matrix, which contains the variability not explained by the linear ANOVA model. Significance of the effect can be estimated by the sum of squares (SSQ) of the elements of the effect matrix **X**_phase_ and its statistical significance may be evaluated by comparing the experimental SSQ with its distribution under the null hypothesis, which can be obtained by permutation tests ^1^. If the particular design term is found to have a significant effect, then interpretation can be carried out by calculating a principal component analysis model of the effect matrix (in the present case, **X**_phase_), which allows evaluating what are the changes in the multivariate experimental profile induced by the different levels of the controlled factor(s).

**Cell culture and treatments**

The SZ95 immortalized human sebaceous gland cell line (Zouboulis et al., 1999), showing morphologic, phenotypic and functional characteristics of normal human sebocytes, was cultured in Sebomed® basal medium (Merk Life Science S.r.l.,Milan, Italy) supplemented with 10% foetal bovine serum (FBS) (Invitrogen, Milan, Italy), 2mM L-glutamine (Invitrogen, Milan, Italy), 100 µg/ml penicillin/streptomycin (Invitrogen, Milan, Italy), 5 ng/ml recombinant human epidermal growth factor (EGF) (Thermo Fisher Scientific, Monza, Italy) and 1 mM CaCl2 in a humidified atmosphere containing 5% CO2 at 37°C. Cell line was routinely tested for Mycoplasma detection. For the experiments, SZ95 sebocytes were starved overnight and maintained without FBS. SZ95 sebocytes were treated with vehicle (0,1 % ethanol), or 1 µM 17β-estradiol (E2) (supplier), 1 µM progesterone (P) (supplier), or the combination of both sex hormones. All experiments were performed at least in triplicate.

**RNA extraction and quantitative real-time RT-PCR**

Total RNA was isolated from SZ95 sebocytes using the Aurum™ Total RNA Mini kit (Bio-Rad Laboratories Srl, Milan, Italy), according to the manufacturer’s instructions. Total RNA samples were stored at -80°C until use. Following DNAse I treatment, cDNA was synthesized using a mix of oligo-dT and random primers and RevertAid™ First Strand cDNA synthesis kit according to the manufacturer’s instructions. Real-time RT-PCR was performed in a total volume of 10 μL with SYBR Green PCR Master Mix (Bio-Rad Laboratories Srl, Milan, Italy) and 200 nM concentration of each primer. The primers used in this study are as follows: GAPDH 5’-TGCACCACCAACTGCTTAGC-3’ (forward), 5’-GGCATGGACTGTGGTCATGAG-3’ (reverse); SREBP1 5’- TTAGAGCGAGCACTGAAC-3’ (forward), 5’- TGGAACTGATGGAGAAGC-3’ (reverse); FASN 5’- GACCGCTTCCGAGATTCC-3’ (forward), 5’- GATGGCAGTCAGGCTCAC -3’ (reverse); FADS2 5’-TGTCTACAGAAAACCCAAGTGG-3’ (forward), 5’-TGTGGAAGATGTTAGGCTTGG-3’ (reverse); SCD1 5’-CATAATTCCCGACGTGGCTTT-3’ (forward), 5’-AGGTTTGTAGTACCTCCTCTGGAACA-3’ (reverse); PPARγ 5’-GCCAAGCTGCTCCAGAAAAT-3’ (forward), 5’- TGATCACCTGCAGTAGCTGCA-3’ (reverse). Reactions were carried out in triplicate using a CFX96 Real Time System (Bio-Rad Laboratories S.r.l.). Melt curve analysis was performed to confirm the specificity of the amplified products. The mRNA expression was normalized to the mRNA expression of GAPDH by the change in the Δ cycle threshold (ΔCt) method and calculated based on 2-ΔCt. Data were represented as mean ± SD of three independent experiments. The values were expressed as relative to the control (vehicle, set as 1). Statistical significance was assessed using paired Student’s t-test. The minimal level of significance was p ≤ 0.05.

**Nile red assay**

At the end of the treatments, SZ95 sebocytes were washed twice with PBS and stained with Nile red (NR) 1 µg/mL solution in for 10 min in a humidified atmosphere containing 5% CO2 at 37°C (Greenspan,P. 1985). Fluorescence intensity was quantified using the fluorescence multi-reader DTX 880 Multimode Detector (Beckman Coulter Srl, Milan, Italy), by setting the excitation (λex) and the emission (λem) wavelengths at 485, and 574 nm, respectively, to detect neutral lipids. Polar lipids were detected in the same cells by setting λex and λem at 535 and 625 nm, respectively (Greenspan,P. 1985). Data were represented as mean ± SD of three independent experiments in hexaplicate. The values were expressed as relative to the control (vehicle, set as 1). Statistical significance was assessed using 2-way Anova followed by Tukey’s multiple comparisons test using GraphPad Prism (GraphPad Software). The minimal level of significance was p ≤ 0.05.
